# THE IMPACT OF CHILDHOOD ADVERSITIES ON INTERPERSONAL AND INSTITUTIONAL DISCRIMINATION IN MIDDLE AND LATE ADULTHOOD

**DOI:** 10.1093/geroni/igae098.3205

**Published:** 2024-12-31

**Authors:** Wenxing Wei

**Affiliations:** Case Western Reserve University, Cleveland, Ohio, United States

## Abstract

**Introduction:**

Research on the impact of childhood adversities on later-life experiences of interpersonal and institutional discrimination is scarce. Drawing on the life course perspective, this study examines how childhood adversities influence interpersonal and institutional discrimination experienced in middle and late adulthood.

**Methods:**

Utilizing the Health and Retirement Study (HRS), this study included a nationally representative sample of 14,268 adults aged 45 and older. Ordinary Least Squares (OLS) regressions were conducted to examine whether childhood adversities were associated with interpersonal and institutional discrimination, controlling for socio-demographic characteristics.

**Results:**

Findings indicate a significant relationship between childhood adversities and both interpersonal and institutional discrimination in later life. Specifically, increased experiences of childhood adversities were associated with higher levels of reported discrimination, underscoring the persistent effects of early adversities on individuals’ later life experiences.

**Discussion:**

This study contributes to the existing literature by focusing on the impact of childhood adversities on discrimination in an age group frequently neglected in studies. It underscores the importance of developing comprehensive prevention and intervention to mitigate the long-term effects of childhood adversities in middle and late adulthood. Additionally, the research identifies directions for future studies to delve into the mechanisms of these associations, to consider a wider range of childhood adversities (e.g., bullying), and to examine discrimination on broader levels (e.g., systemic). These explorations are crucial for the development of more effective support strategies and policy frameworks for individuals affected by childhood adversities and experiencing discrimination.

